# Immunometabolic and Lipidomic Markers Associated With the Frailty Index and Quality of Life in Aging HIV+ Men on Antiretroviral Therapy

**DOI:** 10.1016/j.ebiom.2017.07.015

**Published:** 2017-07-18

**Authors:** Hui-Ling Yeoh, Allen C. Cheng, Catherine L. Cherry, Jacquelyn M. Weir, Peter J. Meikle, Jennifer F. Hoy, Suzanne M. Crowe, Clovis S. Palmer

**Affiliations:** a Department of Infectious Diseases, The Alfred and Monash University, Level 2, Burnet Institute, 85 Commercial Road, Melbourne, VIC 3004, Australia; b Burnet Institute, 85 Commercial Road, Melbourne, VIC 3004, Australia; c Department of Epidemiology and Preventive Medicine, Monash University, Level 6, The Alfred Centre (Alfred Hospital), 99 Commercial Road, Melbourne, VIC 3004, Australia; d School of Physiology, University of the Witwatersrand, 1 Jan Smuts Avenue, Braamfontein, 2000 Johannesburg, South Africa; e Metabolomics Laboratory, Baker Heart and Diabetes Institute, 75 Commercial Road, Melbourne, VIC 3004, Australia; f Department of Microbiology and Immunology, University of Melbourne, Melbourne, Australia

**Keywords:** HIV, Frailty, Aging, Lipids, Metabolism, Monocytes, Immunometabolism, Inflammation, Glut1

## Abstract

Chronic immune activation persists despite antiretroviral therapy (ART) in HIV+ individuals and underpins an increased risk of age-related co-morbidities. We assessed the Frailty Index in older HIV+ Australian men on ART. Immunometabolic markers on monocytes and T cells were analyzed using flow cytometry, plasma innate immune activation markers by ELISA, and lipidomic profiling by mass spectrometry. The study population consisted of 80 HIV + men with a median age of 59 (IQR, 56–65), and most had an undetectable viral load (92%). 24% were frail, and 76% were non-frail. Frailty was associated with elevated Glucose transporter-1 (Glut1) expression on the total monocytes (*p* = 0.04), increased plasma levels of innate immune activation marker sCD163 (OR, 4.8; CI 1.4–15.9, *p* = 0.01), phosphatidylethanolamine PE(36:3) (OR, 5.1; CI 1.7–15.5, *p* = 0.004) and triacylglycerol TG(16:1_18:1_18:1) (OR, 3.4; CI 1.3–9.2, *p* = 0.02), but decreased expression of GM3 ganglioside, GM3(d18:1/18:0) (OR, 0.1; CI 0.0–0.6, *p* = 0.01) and monohexosylceramide HexCerd(d18:1/22:0) (OR, 0.1; CI 0.0–0.5, *p* = 0.004). There is a strong inverse correlation between quality of life and the concentration of PE(36:3) (ρ = − 0.33, *p* = 0.004) and PE(36:4) (ρ = − 0.37, *p* = 0.001). These data suggest that frailty is associated with increased innate immune activation and abnormal lipidomic profile. These markers should be investigated in larger, longitudinal studies to determine their potential as biomarkers for frailty.

## Highlights

• Frailty in older HIV + men is associated with heightened monocyte glycolytic metabolism and innate immune activation.
• Lipid classes including phosphatidylethanolamine, GM3 ganglioside and monohexosylceramide are dysregulated in frailty.
• Reduced quality of life in aging HIV + men is associated with monocyte glycolytic metabolism and plasma lipid dysregulation.

Despite the achievement of long-term survival, people living with HIV have higher rates of age-related comorbidities and frailty (low physiological reserve) compared to their HIV-negative counterparts, for reasons that are currently unknown. We found that immune cells (monocytes) from frail individuals may be incapable of using glucose efficiently, leading to immune dysfunction. Furthermore, we identified a group of lipids in the blood that is associated with frailty. These previously unrecognized factors might underpin age-related comorbidities including frailty in the aging HIV + population. This knowledge may improve our understanding, and management of the health conditions in people aging with HIV.

## 1 Introduction

As the median age of people living with HIV increases globally (UNAIDS, 2013), there is a growing need to identify and manage the compounded effects of HIV and aging. In geriatrics, it is generally accepted that chronological age may not accurately reflect physiologic reserve. Frailty conceptualizes a “biologic age”, describing an overall physiologic vulnerability to stressors. Frailty is not only a predictor of adverse outcomes during aging, it has also been used to individualize treatment goals for chronic comorbidities to improve health outcomes (Blodgett et al., 2016, Strain et al., 2013). Chronically infected and long-term treated HIV+ people have a heightened risk, and possibly premature onset of, a number of non-AIDS comorbidities including cardiovascular disease, non-AIDS cancers, renal and bone disease, liver disease as well as frailty (Deeks, 2011).

The clinical definition of frailty remains controversial, but one validated measure is the Frailty Index, where frailty is the result of an accumulation of health deficits over time, and the severity of frailty can predict adverse health outcomes (Rockwood and Mitnitski, 2007).

In the general population, frailty has been shown to be associated with activation of the innate immune system (Collerton et al., 2012). Similarly, HIV infection is associated with biomarkers of innate immune system activation. sCD14 remains elevated despite effective antiretroviral therapy, and has also been identified as a potential biomarker for non-AIDS mortality risk in HIV+ people (Méndez-Lagares et al., 2013, O'Halloran et al., 2015). Elevated levels of other innate immune activation markers including soluble CD163 (sCD163), which is shed from macrophages via proteolytic cleavage by the sheddase ADAM-17 following an inflammatory stimulus (Etzerodt et al., 2010), and thus is also considered an inflammatory marker, have also been linked with both HIV infection and aging (Hearps et al., 2012, O'Halloran et al., 2015). Recognition of a major role of the innate immune system in driving age-related comorbidities via chronic inflammation and senescent change marks a shift from the traditional focus on adaptive immune dysfunction that is largely responsible for CD4^+^ T cell depletion in untreated HIV infection (Hearps et al., 2014).

Immunometabolism is a rapidly evolving area providing insights into how metabolic changes in immune cells impact immunity and drives inflammatory diseases. Overwhelming evidence indicate specific metabolic demands of immune cells during an infection. In HIV+ individuals, increased glucose metabolic activity in inflammatory CD16+ monocyte subsets was associated with inflammation, and this metabolic activity was not normalized in patients on antiretroviral therapy (Anzinger et al., 2014, Palmer et al., 2014a). This metabolic activity is controlled by the mitochondria and may be perturbed by HIV itself or ART toxicity. The immunometabolic concepts of this study present potential prognostic and therapeutic opportunities. In particular, glucose transporter 1 (Glut1), the major glucose transporter on inflammatory monocytes and macrophages, is overexpressed on inflammatory monocytes/macrophages to maintain activation and produce pro-inflammatory cytokines (Palmer et al., 2014a, Freemerman et al., 2014).

HIV infection and antiretroviral therapy are both associated with significant disturbances in systemic metabolic regulation, including lipids, which play a central role in the host immune response, as previously reviewed (Willig and Overton, 2016). While dyslipidemia is a recognized risk factor for age-related diseases, such as cardiovascular disease, only a handful of studies have investigated the plasma lipid profiles in HIV infection, and these have focused on cardiovascular risk prediction (Wong et al., 2014, Low et al., 2016). There is a paucity of data reflecting the association between dysregulated lipid metabolism with frailty.

Thus, we conducted a comprehensive profiling of key plasma lipid species in older HIV+ men to uncover previously unrecognized lipids that may play a role in the immunopathogenesis of frailty. We sought to investigate the relationship between frailty, innate immune activation, and the immunometabolic and lipidomic profiles of older men with HIV infection.

## 2 Materials and Methods

### 2.1 Study Population

Participants were recruited voluntarily between March and August 2015 from the HIV outpatient clinic and Fairfield House, a sub-acute inpatient facility, at The Alfred hospital, a tertiary care facility in Melbourne, Australia. Individuals were eligible if they were living with HIV, of male sex, aged fifty years or older, able to participate in the study assessments, and were taking combination antiretroviral therapy for at least six months. Approval for this study was obtained from the Alfred Health Human Research Ethics Committee and the Monash University Human Research Ethics Committee (Project No. 41/15), with a signed informed consent provided by all volunteers.

### 2.2 Clinical Assessments

All participants completed a single study visit comprising a standardized interview, clinical frailty assessments, and review of available medical records. Time since HIV diagnosis, previous AIDS diagnoses, nadir CD4^+^ T-cell count, antiretroviral drug history, and anthropometric data from the prior 12 months were collected from medical records. Current comorbidities and medications were self-reported and confirmed by medical records. These included non-AIDS cancers, liver disease, mental health illnesses, arrhythmias, congestive heart failure, venous thromboembolism, hypertension, ischemic heart disease, peripheral vascular disease, chronic respiratory disease, painful arthropathies, osteoporosis, rheumatoid arthritis, cerebrovascular disease, renal disease, neurological diseases, diabetes mellitus and thyroid disorders. Laboratory measures were obtained from blood tests performed on the day of assessment, or from the medical record within six months of study enrollment.

### 2.3 Frailty Assessment

Frailty was assessed using the Frailty Index. The Frailty Index implemented in this study was composed of 37 age-related health variables, previously published, with minor adaptations (Searle et al., 2008). Modifications were the removal of peak flow, shoulder strength and rapid pace due to lack of feasibility of performing these in the clinic. We also added urinary incontinence as a variable, as this was an important functional indicator, and met the five criteria for inclusion into the Frailty Index as a variable (Searle et al., 2008). The number of reported deficits was divided by the number of assessed variables to calculate a final ratio score for each individual ranging from 0 to 1, with 1 indicating the worst frailty. Comorbidities were assessed as “present” if self-reported and verified within medical records, and “suspected” if detected during the study visit, but with no formal diagnosis or not receiving treatment for the condition. Participants with a Frailty Index score > 0.25 were considered “frail” and ≤ 0.25 “non-frail” (Rockwood et al., 2007).

### 2.4 Quality of Life

Quality of life was measured using the RAND-36 measure of health-related quality of life, a 36-item survey used commonly in the HIV + population. Quality of life was calculated as a score from 0 to 100, as previously described (Hays and Morales, 2001). Higher values indicate higher self-reported quality of life.

### 2.5 Peripheral Blood Mononuclear Cell (PBMC) Preparation

Thirty milliliters of whole blood was collected in EDTA anticoagulant and processed within 2 h of collection to minimize cell activation or cytokine production. PBMCs were purified from whole blood using Ficoll-Paque Plus (GE Healthcare, Uppsala, Sweden) or Lymphoprep (Axis-Shield, Oslo, Rodel, Norway). PBMCs were cryopreserved in 10% dimethyl sulfoxide in RPMI medium as previously described (Palmer et al., 2014b).

### 2.6 Glucose Transporter-1 Expression and Mitochondrial Function

Glycolytic activation was analyzed by quantifying Glut1 surface expression on cryopreserved PBMCs using flow cytometry as previously described (Palmer et al., 2014a). Briefly, PBMCs were stained with fluorescently labeled anti-CD3/CD14/CD16 monoclonal antibodies. Cell surface Glut1 was detected using Glut1 antibody [MAB1418 clone (R&D Systems, Minneapolis, Minnesota, USA)] conjugated with FITC. Cells were stained and acquired as previously described (Palmer et al., 2014a).

To measure mitochondrial membrane potential, PBMCs were stained as above and pellets resuspended in 100 μL of 1 × PBS containing 2 μL of 1 μM DiOC_6_(3), a fluorescent lipophilic dye that is selective for mitochondria. Cells were incubated in the dark at 37 °C for 30 min, washed twice with 0.5% BSA in 1 × PBS, and resuspended in 300 μL of 1 × PBS.

Samples were analyzed immediately by flow cytometry or stored in the dark at 4 °C for up to 30 min before data acquisition.

### 2.7 T-lymphocyte Immune Activation

To measure T cell activation, PBMCs were stained with fluorescently labeled anti-CD3-FITC/CD4-PerCPCy5.5/CD8-APCH7/HLA-DR-PE-Cy7/CD38-PE monoclonal antibodies as previously described (Palmer et al., 2014b).

### 2.8 Data Acquisition

Data were acquired using a FACS Canto II machine (BD Biosciences, San Jose, CA, USA), collecting at least 200,000 events per sample. CD14 and CD16 expression were used to define monocyte subsets: classical (CD14^++^ CD16^−^), intermediate (CD14^++^ CD16^+^) and non-classical (CD14^+^ CD16^++^) (Ziegler-Heitbrock et al., 2010). CD4 and CD8 expression were used to define T-cell subsets as previously described (Palmer et al., 2014b).

To ensure consistency in the inter-run background signal for markers of interest, we monitored the voltage and compensation settings determined by CompBeads (BD Biosciences, San Jose, California, USA). Positivity for Glut1 and DiOC_6_(3) fluorescence was determined using a gate that included 1.0 (± 0.1)% of unstained or isotype-control stained cells. The difference of expression between the geometric mean in the unstained or isotype cells and the Glut1 and DiOC_6_(3) expression was calculated to obtain the mean fluorescence intensity (MFI). MFI and percentage of cells positive for each marker were calculated for each monocyte and T-cell subset.

### 2.9 Measurement of Soluble Markers in Plasma

Plasma was thawed on ice and used in commercially available ELISA to measure sCD163 (Trillium Diagnostics, Bangor, Maine, USA) and sCD14 (R&D Systems, Minneapolis, Minnesota, USA). Assays were performed according to the manufacturers' specifications. Biomarker concentrations were calculated using ELISA Analysis (elisakit.com PTY LTD) software to generate four-parameter logistic regression standard curve.

### 2.10 Lipid Extraction and Analysis

Lipids were extracted from plasma samples as described previously (Weir et al., 2013). Briefly, 10 μL of plasma and 10 μL of internal standard mixture was combined with 20 volumes of chloroform:methanol (2:1). The mixture was vortexed and sonicated. The supernatant was removed and dried in a 96-well plate in a speed-vac. Samples were reconstituted in 50% water saturated butanol and 50% methanol with 10 mM ammonium formate before analysis by LC ESI-MS/MS. Samples were analyzed as described previously (Weir et al., 2013), with the following minor modifications. Solvent A 60% H_2_O, 20% MeOH, 20% THF, Solvent B 5% H_2_O, 20% MeOH, 75% THF, both with 10 mM ammonium formate. In total 31 lipid classes covering 327 subspecies were quantified in plasma from each study participant.

### 2.11 Statistical Analyses

The distribution of continuous outcome variables was explored and transformed to approximate normality as appropriate. Analyses were performed using Stata Version 13.1, and graphs were created using GraphPad Prism 6.0. Association between categorical variables and frailty status was evaluated using Fisher's exact test. Association between continuous variables and frailty status was evaluated using the unpaired Student's *t*-test or Mann–Whitney *U* test, as appropriate. Simple logistic regression analyses were performed to examine associations between frailty (Frailty Index score > 0.25) and laboratory markers. Multivariable logistic regression models were then adjusted for age, most recent CD4^+^ T-cell count, presence of hepatitis B (HBV) or C co-infection (HCV), a history of AIDS and CD4:CD8 ratio, as these variables are important HIV-related considerations. Importantly, some, but not all, medical comorbidities counted in this study were also specific variables in the Frailty Index assessment, which would result in a significant overlap between the number of medical comorbidities and frailty status. Thus, an individual's total number of medical comorbidities, and subsequently, the variable of having four or more medical comorbidities were not used in the multivariable regression analyses against frailty, as these would be significant confounding factors. However, hepatitis B and C co-infections were important HIV-associated comorbidities that were not assessed in the Frailty Index, and therefore able to be included in the multivariable regression analyses.

For the hierarchical clustering analysis of lipid expression, unpaired Student's *t*-test and Mann-Whitney *U* test (where appropriate) were performed to identify differential lipids by comparing the means or medians of lipids between the HIV + frail and non-frail groups. Based on *p*-values < 0.05, differential lipids were mapped onto heat-map to find micro patterns. The rows are ordered based on the order of the hierarchical clustering. To assess the similarity of differential lipids between two groups, a distance or score was computed. The heat map was generated using Euclidean distance as the default distance method and complete linkage as the agglomeration method. The heat map shows row dendrogram (hierarchical tree) for differential lipids on one of the axis for the outcomes (HIV + frail & non-frail) on the second axis. Correspondence factor analysis was conducted using R language based package available at The Comprehensive R Archive Network.

## 3 Results

### 3.1 Study Population

The study cohort comprised 100 men with HIV infection, of whom 93 completed the clinical frailty assessment and samples were available for 80 men for laboratory analysis of immunometabolic markers and for 77 men for lipidomic profiling. This study population of 80 older men had a median age of 59 years (IQR, 56–65) and were predominantly Caucasian (95%), with most patients having a preserved CD4^+^ T-cell count (mean ± SD, 621 ± 317 cells/μL) and suppressed peripheral HIV replication (92%) (see Table 1 for detailed cohort demographics).

**Table 1:** Characteristics of the study population, and the non-frail and frail subpopulations.

Characteristic	Study population	Non-frail	Frail	*p*†
N	80	61	19	
Age, median (IQR)	59 (56–65)	59 (56–65)	61 (54–70)	0.22
Caucasian race, *n* (%)	76 (95)	59 (97)	17 (89)	0.24
Pack/years smoking, median (IQR)	9 (0 − 30)	5 (0–25)	12 (0–43)	0.21
Alcohol consumption > 2 standard drinks per day, *n* (%)	14 (18)	10 (16)	4 (21)	0.73
Currently employed, *n* (%)	36 (45)	30 (49)	6 (32)	0.20
Intravenous drug use (ever), *n* (%)	7 (9)	4 (7)	3 (16)	0.26
BMI, mean ± SD	25 ± 3	25 ± 3	25 ± 4	0.94
Waist circumference, mean ± SD	96 ± 11	95 ± 10	99 ± 14	0.12
≥ 4 comorbidities, *n* (%)	24 (30)	8 (13)	16 (84)	< 0.001⁎
≥ 5 non-ART medications, *n* (%)	43 (54)	27 (44)	16 (84)	0.003⁎
Depression/anxiety, *n* (%)	27 (34)	19 (31)	8 (42)	0.41
Osteoporosis, *n* (%)	12 (15)	5 (8)	7 (37)	0.01⁎
Serious non-AIDS events, *n* (%)	34 (43)	22 (36)	12 (63)	0.06
- Cardiovascular disease	18 (23)	12 (20)	6 (32)	0.35
- Decompensated liver disease	6 (8)	3 (5)	3 (16)	0.14
- Type 2 diabetes mellitus	14 (18)	8 (13)	6 (32)	0.09
- Non-AIDS defining cancer	6 (8)	3 (5)	3 (16)	0.14
- Stroke	10 (13)	4 (7)	6 (32)	0.01⁎
Current CD4^+^ cell count (cells/μL), mean ± SD	621 ± 317	647 ± 320	538 ± 300	0.28
Nadir CD4^+^ cell count (cells/μL), median (IQR)	159 (40–266)	180 (39–270)	100 (60–240)	0.53
CD4:CD8 ratio, median (IQR)	0.7 (0.5–1.0)	0.7 (0.5–1.0)	0.6 (0.4–1.3)	0.68
Detectable HIV-viral load, *n* (%)	6 (8)	5 (8)	1 (5)	1.00
History of AIDS, *n* (%)	36 (45)	23 (38)	13 (68)	0.03⁎
Hepatitis B virus co-infection, *n* (%)	4 (5)	3 (5)	1 (5)	0.67
Hepatitis C virus co-infection, *n* (%)	9 (11)	6 (10)	3 (16)	0.44
Time since diagnosis of HIV (months), median (IQR)	243 (124–308)	227 (122–288)	267 (170–328)	0.53
Duration of ART (months), median (IQR)	189 (88–249)	188 (86–244)	198 (105–269)	0.52
ART initiation before 1996, *n* (%)	26 (33)	19 (31)	7 (37)	0.78
Exposure to early nucleoside analogues^a^; ever, *n* (%)/duration (years), median (IQR)	53 (66)/7 (0 − 12)	39 (64)/7 (0–12)	14 (74)/8 (0 − 13)	0.58/0.57
Exposure to protease inhibitors; ever, *n* (%)/duration (years), median (IQR)	54 (68)/7 (0–24)	39 (64)/7 (0 − 21)	15 (79)/12 (0–27)	0.27/0.32
%CD4^+^ CD38^+^ HLA-DR^+^, median (IQR)	1.0 (0.6–1.4)	1.1 (0.7–1.4)	0.8 (0.4–1.4)	0.16
%CD8^+^ CD38^+^ HLA-DR^+^, median (IQR)	2.3 (1.2–4.7)	2.5 (1.4–4.3)	1.6 (0.8–8.7)	0.44
sCD163 level, ng/mL, median (IQR)	2.8 (1.9–3.8)	2.6 (1.8–3.5)	3.6 (2.1–5.9)	0.01⁎
sCD14 level, pg/mL, median (IQR)	6235 (5203–7379)	5927 (5178–7006)	7183 (5896–8680)	0.03⁎
Glut1 MFI on total monocytes, median (IQR)	88 (66–117)	79 (63–115)	101 (78–159)	0.04⁎

Abbreviations: ART, antiretroviral therapy; BMI, body mass index; MFI, mean fluorescence intensity.

† *p*-Value calculated using the Fisher's exact test, unpaired Student's *t*-test or Mann-Whitney *U* test (where appropriate).

⁎ *p*-values < 0.05.

a Early nucleoside analogues were defined as zidovudine, zalcitabine, stavudine and didanosine.

Based on the Frailty Index, 19 (24%) were frail, and 61 (76%) non-frail. The overall median (IQR) Frailty Index score was 0.14 (0.07–0.23). The median (IQR) Frailty Index score for the frail population was 0.39 (0.36–0.44) and for the non-frail population 0.10 (0.06–0.17).

### 3.2 Frailty is Associated With Increased Plasma Levels of Innate Immune Activation Markers

**Fig. 1:**
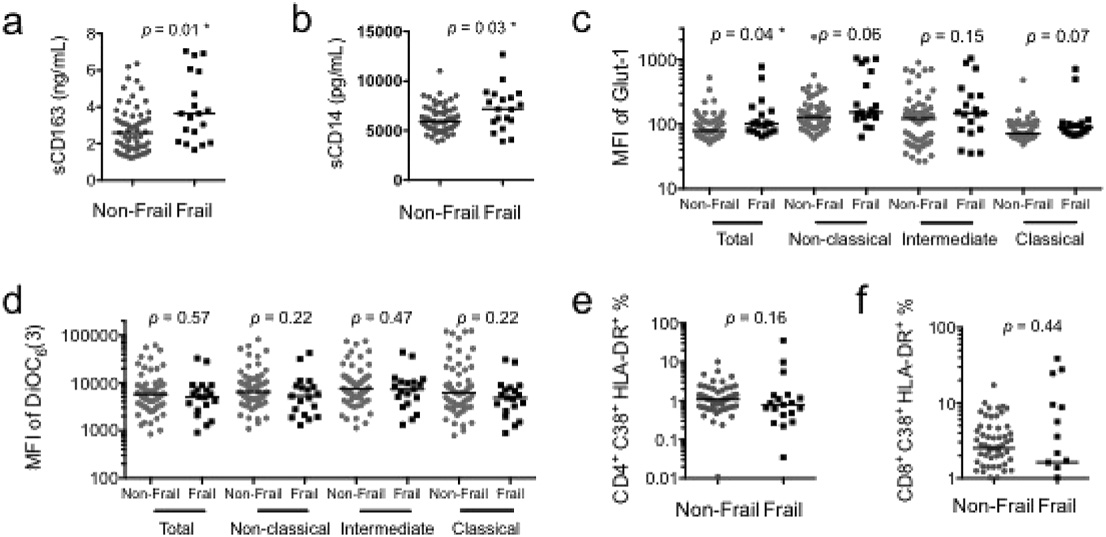
Innate immune activation and metabolic dysregulation are evident in frail HIV-infected men. Plasma from non-frail and frail individuals was examined for (a) sCD163 and (b) sCD14. Monocytes from non-frail and frail individuals were examined for (c) the MFI of Glut1 and (d) the MFI of DiOC_6_(3) on each monocyte subpopulation. T-cells of non-frail and frail individuals were examined for the level of immune activation in (e) CD4^+^ T-cells and (f) CD8^+^ T-cells. * denotes *p*-values < 0.05.

Compared to non-frail persons, frail HIV+ men had higher levels of innate immune activation and inflammation marker, sCD163 (median 2.6 ng/mL vs. 3.6 ng/mL, *p* = 0.01) and sCD14 (median 5927 pg/mL vs. 7183 pg/mL, *p* = 0.03) (Fig. 1a and b). On univariable regression analysis, sCD163 was associated with greater odds of frailty (OR, 4.8; CI 1.4–15.9, *p* = 0.01) (Table 2). The association between sCD163 and frailty was independent of age, current CD4^+^ T-cell count, co-infection with HBV or HCV, a history of AIDS, or CD4:CD8 ratio (OR, 7.5; CI 1.4–39.7, *p* = 0.02) (Table 2). sCD14 was also associated with a noticeable positive relationship with frailty (OR, 11.4; CI 1.2–109.1, *p* = 0.04), and after correcting for previously described variables (OR, 8.4; CI 0.7–96.2, *p* = 0.09) (Table 2).

**Table 2:** Multivariable analysis of immunometabolic markers and lipid subclasses associated with frailty in older HIV + men.

	Unadjusted			Adjusted^a^		
	OR	(95% CI)	*p*	OR	(95% CI)	*p*
Innate immune activation						
sCD163 level, ng/mL	**4.8**	**(1.4, 15.9)**	**0.01**⁎	**7.5**	**(1.4, 39.7)**	**0.02**⁎
sCD14 level, pg/mL	**11.4**	**(1.2, 109.1)**	**0.04**⁎	8.4	(0.7, 96.2)	0.09⁎⁎

Metabolic dysregulation (Glut1 MFI)						
Monocytes						
Total monocyte population	**2.6**	**(1.0, 6.8)**	**0.04**⁎	**3.8**	**(1.2, 12.5)**	**0.03**⁎
Non-classical (CD14^+^ CD16^++^)	**2.2**	**(1.1, 4.4)**	**0.03**⁎	**3.0**	**(1.3, 6.9)**	**0.01**⁎
Intermediate (CD14^++^ CD16^+^)	1.5	(0.9, 2.6)	0.17	2.0	(1.0, 3.9)	0.05⁎⁎
Classical (CD14^++^ CD16^−^)	2.9	(1.0, 9.1)	0.06⁎⁎	4.2	(1.0, 18.3)	0.06⁎⁎
T-cells						
CD4^+^	0.6	(0.2, 1.6)	0.29	0.6	(0.2, 1.9)	0.38
CD8^+^	1.2	(0.8, 1.9)	0.33	1.4	(0.8, 2.2)	0.21

Mitochondrial dysfunction (DiOC_6_(3) MFI)						
Monocytes						
Total monocyte population	0.8	(0.5, 1.4)	0.43	0.7	(0.4, 1.3)	0.29
Non-classical (CD14^+^ CD16^++^)	0.7	(0.4, 1.2)	0.20	0.6	(0.3, 1.1)	0.11
Intermediate (CD14^++^ CD16^+^)	0.8	(0.4, 1.4)	0.43	0.7	(0.4, 1.4)	0.31
Classical (CD14^++^ CD16^−^)	0.7	(0.4, 1.1)	0.13	0.6	(0.4, 1.2)	0.14

Adaptive immune activation						
% CD4^+^ CD38^+^ HLA-DR^+^	0.9	(0.5, 1.4)	0.53	0.7	(0.4, 1.3)	0.26
% CD8^+^ CD38^+^ HLA-DR^+^	1.1	(0.6, 1.8)	0.80	1.0	(0.5, 1.9)	0.93

Lipid subtype expression						
PE(36:3), pmol/mL	**5.1**	**(1.7, 15.5)**	**0.004**⁎	**7.2**	**(1.9, 28.2)**	**0.004**⁎
PE(36:4), pmol/mL	**8.8**	**(2.0, 38.6)**	**0.004**⁎	**11.5**	**(2.0, 68.0)**	**0.007**⁎
TG(16:1_18:1_18:1), pmol/mL	**3.4**	**(1.3, 9.2)**	**0.02**⁎	3.5	(1.0, 12.0)	0.05⁎⁎
GM3(d18:1/18:0), pmol/mL	**0.1**	**(0.0, 0.6)**	**0.01**⁎	**0.1**	**(0.0, 0.7)**	**0.03**⁎
HexCerd(d18:1/22:0), pmol/mL	**0.1**	**(0.0, 0.5)**	**0.004**⁎	**0.1**	**(0.0, 0.5)**	**0.01**⁎
HexCerd(d18:1/24:0), pmol/mL	**0.1**	**(0.0, 0.5)**	**0.01**⁎	**0.1**	**(0.0, 0.6)**	**0.02**⁎

Abbreviations: MFI, mean fluorescence intensity.

Each line represents a separate regression model for frailty. Bold represents significant p values < 0.05.

a Each multivariable regression model was adjusted for age, current CD4^+^ T-cell count, hepatitis B or C co-infection, a history of AIDS and CD4:CD8 ratio.

⁎ *p*-values < 0.05.

⁎⁎ *p*-values < 0.10.

### 3.3 Frailty is Associated With Increased Monocyte Glut1 Expression but not Mitochondrial Dysfunction

Compared to non-frail persons, frail individuals had higher Glut1 expression on the total monocyte population (median MFI 79 vs. 101, *p* = 0.04) (Fig. 1c). A similar trend was observed in all monocyte subsets, but did not reach significance (Fig. 1c). Increased Glut1 expression on total and non-classical monocytes was associated with frailty (OR, 2.6; CI 1.0–6.8, *p* = 0.04; OR, 2.2; CI 1.1–4.4, *p* = 0.03, respectively) (Table 2). This association remained independent of age, current CD4^+^ T-cell count, co-infection with HBV or HCV, a history of AIDS, and CD4:CD8 ratio in both total and non-classical monocyte populations (OR, 3.8 CI 1.2–12.5, *p* = 0.03; OR, 3.0; CI 1.3–6.9, *p* = 0.01, respectively) (Table 2).

Mitochondrial membrane potential (DiOC_6_(3) MFI), did not differ between non-frail and frail persons in any monocyte population (Fig. 1d; Table 2).

### 3.4 Frailty is not Associated With CD4^+^ or CD8^+^ T Cell Activation

We observed no difference between levels of immune activation (CD38/HLA-DR co-expression) in CD4^+^ and CD8^+^ T-cells from frail and non-frail individuals (Fig. 1e and f).

### 3.5 Lipidomic Profiling Shows Significant Lipid Dysregulation in Frailty

**Fig. 2:**
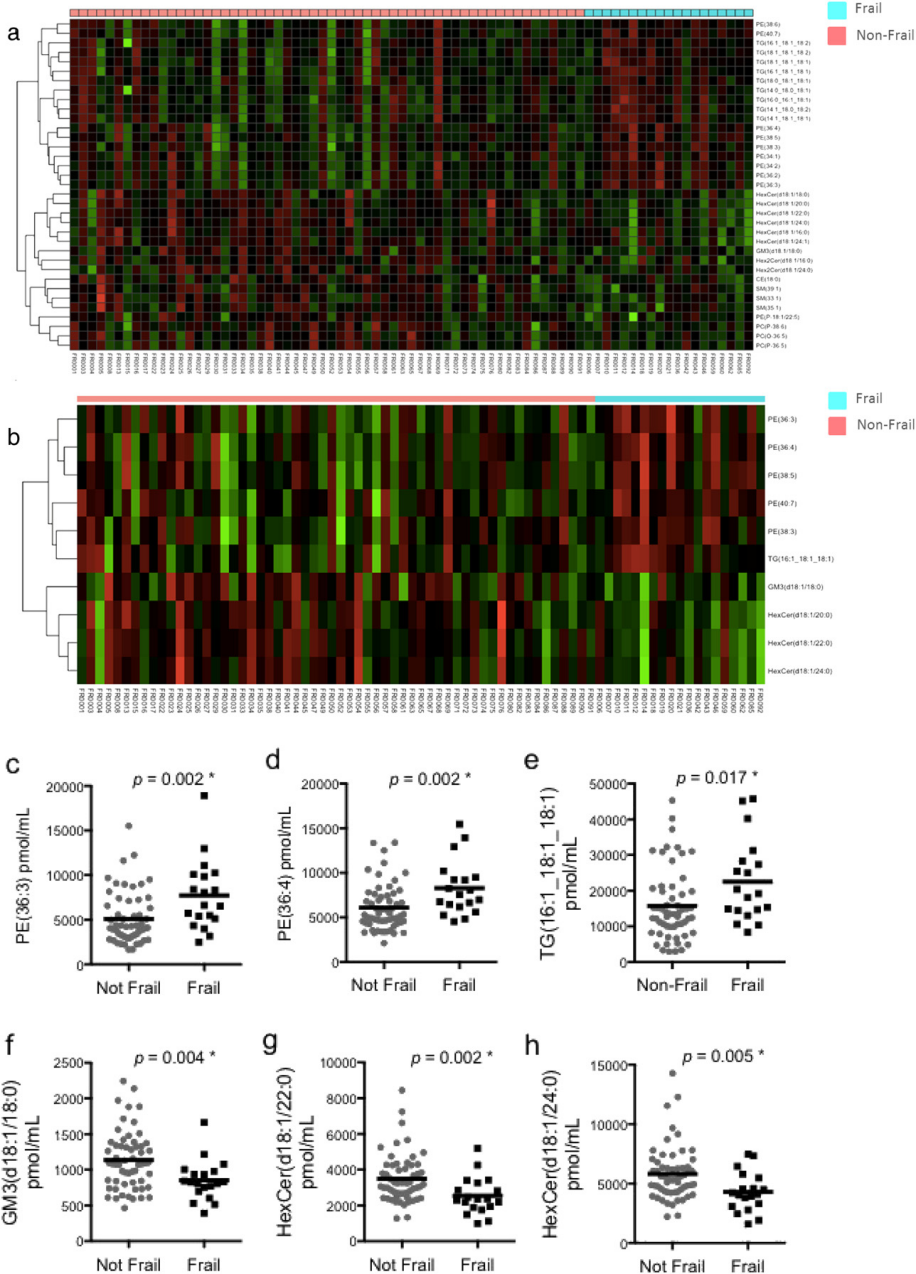
Quantification of lipids in frail HIV + older men. (a) Hierarchical clustering showing 11 classes of differentially expressed lipids among frail and non-frail HIV + older men. (b) Top 10 differentially expressed lipids among frail and non-frail HIV + older men. (c–h) Dot plots showing highly expressed regulated individual lipid species. * denotes *p*-values < 0.05. (For interpretation of the references to color in this figure, the reader is referred to the web version of this article.)

Fig. 2a shows the hierarchical clustering and heat map indicating greater (red) and less (green) differentially expressed plasma lipids in frail and non-frail individuals.

Of the thirty-one lipid classes (covering 327 lipid subspecies) analyzed, 11 classes (PE: phosphatidylethanolamine, TG: triacylglycerol, HexCer: monohexosylceramide, GM3: GM3 ganglioside, Hex2Cer: dihexosylceramide, CE: cholesterol ester, SM: sphingomyelin, PC: phosphatidylcholine, PE(P): alkylphosphatidylethanolamine, PC(P): alkenylphosphatidylcholine, PC(O): alkylphosphatidylcholine) were differentially regulated between frail and non-frail participants (Fig. 2a).

The top ten differentially regulated lipids belonged to only four groups (phosphatidylethanolamine, triacylglycerol, GM3 ganglioside, monohexosylceramide) (Fig. 2b). The phosphatidylethanolamine and triacylglycerol classes were over-expressed in frail individuals compared to non-frail individuals (Fig. 2c–e), while the GM3 ganglioside and monohexosylceramides were under-expressed in frail persons (Fig. 2f–h).

When adjusted for age, current CD4^+^ T-cell count, co-infection with HBV or HCV, a history of AIDS, and CD4:CD8 ratio, several lipid species belonging to the monohexosylceramide, GM3 ganglioside, sphingomyelin, phosphatidylethanolamine and alkylphosphatidylethanolamine classes were independently associated with frailty (*p* < 0.05, Table 2).

### 3.6 Quality of Life

There was a negative correlation between overall quality of life and innate immune activation marker and inflammation sCD163 (ρ = − 0.27; *p* = 0.02), and with the lipid biomarkers PE(36:3) (ρ = − 0.33; *p* = 0.004) and PE(36:4) (ρ = − 0.37; *p* = 0.001). Quality of life was positively correlated with GM3, a lipid species under-expressed in frailty GM3(d18:1/18:0) (ρ = 0.29; *p* = 0.01). There were negatively correlated trends between overall quality of life and plasma innate immune activation marker sCD14 (ρ = − 0.22; *p* = 0.05) and metabolic activation marker Glut1 expression on the total monocyte population (ρ = − 0.19; *p* = 0.10). Overall quality of life was not associated with T-lymphocyte activation or with mitochondrial dysfunction (Suppl. Table 1).

**Fig. 3:**
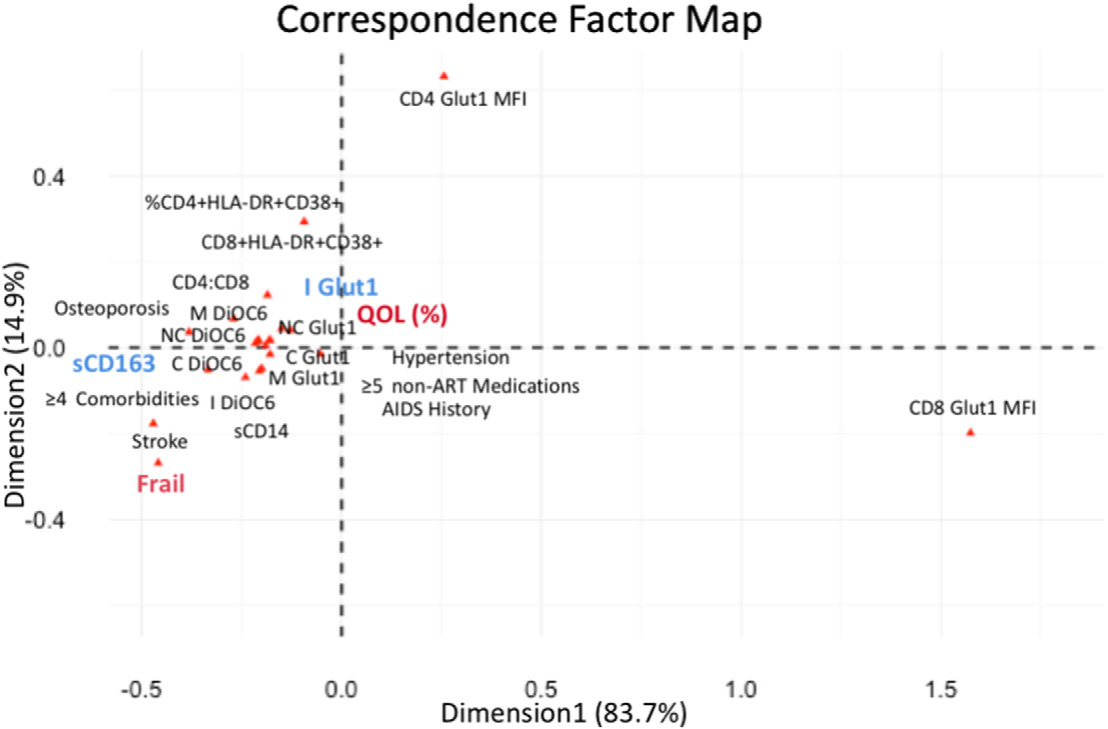
Correspondence factor map showing the inter-relationship between markers of immune activation, inflammation, immunometabolic parameters, and clinical variables in older HIV + men on antiretroviral therapy. Abbreviations: M, total monocytes; C, classical monocytes; I, intermediate monocytes; NC, non-classical monocytes; DiOC6, DiOC_6_(3).

A correspondence factor map was constructed to highlight the inter-relationship between markers of immune activation, immunometabolism and key clinical parameters. As expected given the results above, sCD14 (marker of innate immune activation) and sCD163 (marker of innate immune activation and inflammation) clustered with frailty, comorbidities, and quality of life. Clustering of key clinical variables with metabolic markers on monocyte populations was more evident than metabolic markers on CD8^+^ and CD4^+^ T cells (Fig. 3).

### 3.7 Relationships Between the Parameters of Immune Activation, Metabolic Activity and Lipid Expression

**Fig. 4:**
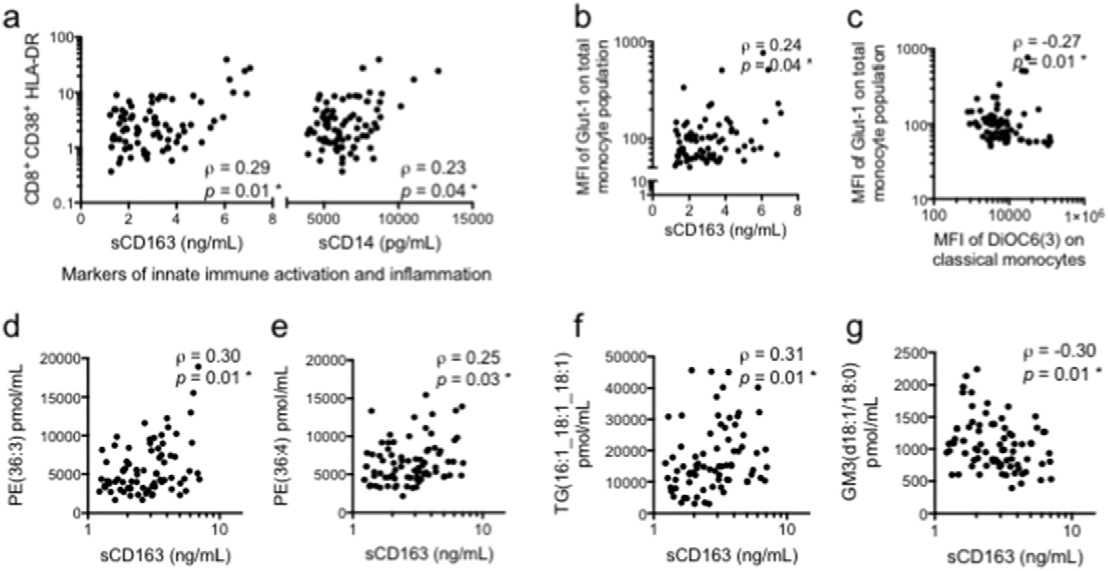
Relationship between markers. (a) Markers of innate immune activation (sCD163 and sCD14) are positively correlated with increased immune activation of CD8^+^ T-cells. (b) sCD163 is positively correlated with increased metabolic activation (Glut-1 MFI) in the total monocyte population. (c) Inverse correlation between metabolic activation and mitochondrial membrane potential. Inflammatory marker, sCD163, is positively correlated with (d) PE(36:3), (e) PE(36:4) and (f) TG(16:1_18:1_18:1), and negatively correlated with (g) GM3(d18:1/18:0).

Markers of adaptive immune activation measured by CD38 and HLA-DR co-expression on CD8^+^ T-cells correlated positively with plasma markers of innate immune activation, sCD163 (ρ = 0.29, *p* = 0.01) and sCD14 (ρ = 0.23, *p* = 0.04) (Fig. 4a), but not with phenotypic markers of metabolic activation on monocyte subsets (Suppl. Table 2a). The percentage of CD4^+^ T-cells co-expressing CD38 and HLA-DR did not correlate with any markers of innate immune activation or with metabolic activation and mitochondrial dysfunction of monocyte subsets (Suppl. Table 2a).

Levels of sCD163, a plasma marker of innate immune activation and inflammation, correlated with Glut1 expression in the total population of monocytes (ρ = 0.24; *p* = 0.04) (Fig. 4b), but not with the metabolic activity of individual subsets of monocytes or with markers of mitochondrial dysfunction (Suppl. Table 2a).

Expression of the mitochondrial functional marker DiOC_6_(3) on classical monocytes, the majority monocyte subset in blood, was inversely correlated with Glut1 expression on total monocytes (ρ = − 0.27; *p* = 0.01) (Fig. 4c), and classical monocytes (Suppl. Table 2b). A similar inverse trend was observed in non-classical and intermediate monocytes (Suppl. Table 2b).

Levels of lipids including PE(36:3), PE(36:4) and TG(16:1_18:1_18:1) correlated significantly with sCD163 (Fig. 4d–f). GM3(d18:1/18:0) correlated inversely with sCD163 (Fig. 4g). Markers of adaptive immune activation, co-expression of CD38 and HLA-DR on CD4^+^ and CD8^+^ T-cells correlated with expression of PE(36:3) (Suppl. Table 2c). However, there were no significant correlations between lipidomic markers and other markers of immune activation, metabolic activation and mitochondrial dysfunction of monocyte subsets (Suppl. Table 2c).

## 4 Discussion

We investigated the global expression patterns of plasma lipids and the immunometabolic characterization of monocyte subpopulations in older HIV+ men on antiretroviral therapy. Our findings demonstrate that frailty in HIV+ men is associated with activation of the innate immune system, and metabolic activation of monocytes. Secondly, frail HIV+ individuals have a distinct lipidomic profile, with the lipid subclasses phosphatidylethanolamine and triacylglycerol associated with frailty and poorer quality of life in HIV+ individuals. Finally, we observed no evidence in this cohort that changes in monocyte mitochondrial membrane potential is associated with frailty.

Levels of sCD14 (marker of innate immune activation), sCD163 (reflecting both innate immune activation and a response to inflammation) and cell surface levels of Glut1 on total monocytes were associated with frailty in our study. This is consistent with the accepted view that persistent activation of the innate immune system plays a central role in the pathogenesis of age-related comorbidities including cardiovascular disease and osteoporosis (Deeks, 2011). Frailty was strongly associated with markers of innate immune activation, measured by plasma levels of sCD163 and to a lesser extent sCD14, which are both shed from the surface of activated monocytes and macrophages. In HIV+ people, sCD163 has been associated with risk of obesity and neurocognitive impairment (Conley et al., 2015, Burdo et al., 2013). Unchanged levels of sCD14 have also been identified as a predictor of serious non-AIDS events (Sunil et al., 2016), but the precise mechanisms of this association are unknown. Our data showing association between frailty and sCD14 are consistent with the findings of the Multicenter AIDS Cohort Study (Margolick et al., 2017), despite this group using a distinctly different measurement of frailty to our study, the Frailty Phenotype.

We have also observed that elevated Glut1 on monocytes is associated with high sCD163 plasma levels within this HIV+ population, supporting a model by which chronic HIV infection drives metabolic activation of monocytes in response to inflammatory changes. This results from metabolic reprogramming within monocytes, switching metabolism from predominantly oxidative phosphorylation to glycolysis. This increase in glycolysis is supported by elevated cell surface Glut1 expression, facilitating more glucose entry into the cell to meet its energy requirements during cellular activation and promoting inflammation (Palmer et al., 2014a, Palmer et al., 2016). Indeed, increased expression of Glut1 on macrophages is a feature of cells that produce high levels of pro-inflammatory molecules, such as TNF and IL-6 (Freemerman et al., 2014). It is unknown whether the expression of CD14 and CD163 and the subsequent cleavage of their soluble products (sCD14 and sCD163) are independent of mechanisms that support metabolic reprogramming of monocytes.

We have previously demonstrated that Glut1 expression is up-regulated in monocytes from HIV-infected compared to uninfected individuals, even after a mean of 9.3 years of antiretroviral therapy (Palmer et al., 2014a). Further, expression of Glut1 is elevated on the intermediate subset of monocytes from HIV-infected women with subclinical cardiovascular disease, supporting a possible role for monocyte glucose metabolic reprogramming in the pathogenesis of non-AIDS comorbidities (Butterfield et al., 2017). Our study found an association between frailty and increased Glut1 expression on the total monocyte population, which marks activation of glycolysis.

We did not observe an association between frailty and mitochondrial membrane potential as measured by DiOC_6_(3) fluorescence in monocytes. DiOC_6_(3) is a dye that stains mitochondria in live cells, and is used to measure mitochondrial trans‑membrane potential and mitochondrial integrity, which decrease with mitochondrial dysfunction preceding apoptosis (Macho et al., 1995). The effects of some HIV treatment regimens on mitochondrial dysfunction in adipose tissues are well established (Mccomsey et al., 2013). In a recent study comparing blood samples from HIV+ patients with samples from HIV negative controls, Yu and colleagues demonstrated that HIV infection was associated with an increase in mitochondrial membrane potential in CD4^+^ and CD8^+^ T cells (Yu et al., 2017). Lymphocytes from HIV+ individuals have also been shown to have significant defects in mitochondrial membrane potential compared to HIV negative controls, with monocytes largely unaffected (Perrin et al., 2012). Our findings do not rule out a role for mitochondrial dysfunction in the pathogenesis of frailty. It is plausible that HIV and/or particular antiretroviral therapy regimens may impact parameters of mitochondrial functions, such as mass, structure and efficiency in terms of ATP production, that have not been evaluated here.

It has previously been shown that HIV infection and aging are associated with T cell activation (Cobos Jiménez et al., 2016, Yao et al., 2011). However, we observed no differences in activation measured by CD38 and HLA-DR overexpression between frail and non-frail HIV-infected men. Our findings are consistent with other studies where markers of T cell activation did not predict the development of serious non-AIDS events (Tenorio et al., 2014). Likewise, we observed no association between frailty and metabolic activation marked by Glut1 levels on T cells.

Dyslipidemia is well documented in virologically suppressed HIV positive patients on antiretroviral therapy (Schulte-Hermann et al., 2016). In fact, HIV infection is associated with significantly deranged plasma lipid profiles compared to HIV negative controls (Wong et al., 2014). Wong and colleagues have identified key lipid classes, in particular, the GM3 ganglioside and monohexosylceramide subclasses, which were negatively associated with HIV infection (Wong et al., 2014), however their clinical relevance in the context of age-related co-morbidities in HIV infection is not well established. In our study, these lipid classes were negatively associated with frailty, and the GM3 ganglioside subclass was positively correlated with quality of life. Our study also found that the lipid classes, triacylglycerol and phosphatidylethanolamine, that have been shown to predict cardiovascular disease in HIV+ people (Wong et al., 2014), were in our study significantly associated with frailty and poorer quality of life. Despite this, there are only very limited data demonstrating a relationship between precise lipid species and age-related non-AIDS morbidity.

Furthermore, the specific role of these lipid subclasses on immune cells is uncertain, and may vary according to lipid subtypes. Evidence suggests that depending on the subtype, some phosphatidylethanolamines have a pro-inflammatory role in activating macrophages (Guo et al., 2015), and appear to be elevated in several cancers (Perrotti et al., 2016). We also observed that sCD163 was positively correlated with the phosphatidylethanolamine and triacylglycerol classes, but inversely with the GM3 ganglioside class correlated inversely with sCD163. GM3 gangliosides can have both pro-inflammatory and anti-inflammatory effects (Wentworth et al., 2016, Kim et al., 2014), similar to expression of CD163.

The role of the monohexosylceramide lipid subclass in the pathogenesis of age-related comorbidities in HIV infection has not previously been investigated. We observed an association between reduced monohexosylceramide plasma lipid levels and frailty. The observed correlation between monohexosylceramide lipid class and GM3 ganglioside was expected, as they are both ceramide metabolites.

The precise link between lipids like phosphatidylethanolamine and monocyte immune activation has not been established. However, phosphatidylethanolamine is a ligand for the phospholipid receptor CD300a that belongs to the CD300 family of paired activating/inhibitory receptors that are highly expressed on myeloid cells including monocytes (Carnec et al., 2015). Pro-inflammatory intermediate and non-classical monocytes have high cell surface levels of CD300a than classical monocytes (Zenarruzabeitia et al., 2016). Furthermore, like Glut1, CD300a is a LPS-responsive hypoxia-inducible gene, regulated by hypoxic environments to induce monocyte/macrophage activation and pro-inflammatory responses (Raggi et al., 2014). Since CD300a may be regulated by PI3Kinase, which also regulates Glut1 and glycolysis, further investigation is warranted to determine if CD300a centrally connects inflammatory lipids, with immune cellular glucose metabolism and inflammatory-mediated responses, and if this is central to frailty pathogenesis. Adoption of a pro-inflammatory monocyte state in diseases has generally centered on the relationship with plasma levels of inflammatory cytokines such as TNF-α, IL-6 and IL-1β. Less is known about the direct impact of perturbed plasma lipid profiles on immune cell metabolism, and how this interaction influences innate and adaptive immune activation, and the course of diseases. Further work is warranted to establish such associations.

This study demonstrates associations between frailty, markers of monocyte glucose metabolic activation and plasma lipid levels. Furthermore, we observed an association between pro-inflammatory lipids, the inflammatory marker sCD163 and quality of life in HIV+ older men on antiretroviral therapy. The limitations of the study include its modest sample size and cross sectional design. Furthermore, lipid profiles are affected by the fasting state and dietary intake, which were not able to be considered in this study. Nonetheless, we have observed clear differences between frail and non-frail participants. Our work identified multiple biomarkers that should now be investigated in larger, longitudinal studies as possible predictors for the development of frailty and its severity. Demonstration of changes in some or all of these biomarkers prior to the onset of frailty could provide a basis for future preventative interventions, and be used to evaluate the effectiveness of anti-inflammatory therapies.

## Funding Sources

This group received a grant from Merck Sharp & Dohme for the materials of this study. The funding sources had no role in designing the study, in collecting, analyzing, interpreting or reporting the data, or in deciding to submit the article for publication. C.S.P. is a recipient of the Australian Centre for HIV and Hepatitis Virology Research (ACH^2^) grant, and a 2010 developmental grant (CNIHR) from the University of Washington Center for AIDS Research (CFAR), an NIH funded program under award number AI027757 which is supported by the following NIH Institutes and Centers (NIAID, NCI, NIMH, NIDA, NICHD, NHLBI, NIA). Some assays employed in this study were developed under these funding schemes. There was no other funding source for this study.

## Appendix A Supplementary data

Supplementary material Image 1
