# Supplementary material for: Immunometabolic and Lipidomic Markers Associated With the Frailty Index and Quality of Life in Aging HIV+ Men on Antiretroviral Therapy
Source: eBioMedicine. 2017 Jul 18;22:112–21. doi: 10.1016/j.ebiom.2017.07.015 (PMC5552224; doi:10.1016/j.ebiom.2017.07.015)
Supplement: Supplementary file 1 — Supplementary material [file mmc1.pdf]

Supplementary Tables

Supplementary Table 1
Correlational analysis of quality of life and immunometabolic markers.

	**Total quality of life**	
	ρ	*p*
**Innate Immune Activation**		
sCD163 level, ng/mL	**-0.27**	**0.02 ***
sCD14 level, pg/mL	-0.22	0.05 **
**Metabolic Dysregulation (Glut1 MFI)**		
Monocytes		
Total monocyte population	-0.19	0.10
Non-classical ($CD14^{+}$ $CD16^{++}$)	-0.21	0.07 **
Intermediate ($CD14^{++}$ $CD16^{+}$)	-0.10	0.40
Classical ($CD14^{++}$ $CD16^{-}$)	-0.22	0.05 **
**Mitochondrial Dysfunction ($DiOC_6(3)$ MFI)**		
Monocytes		
Total monocyte population	0.06	0.63
Non-classical ($CD14^{+}$ $CD16^{++}$)	0.12	0.31
Intermediate ($CD14^{++}$ $CD16^{+}$)	0.08	0.51
Classical ($CD14^{++}$ $CD16^{-}$)	0.11	0.35
**Adaptive Immune Activation**		
% $CD4^{+}$ $CD38^{+}$ HLA-DR^{+}	0.04	0.73
% $CD8^{+}$ $CD38^{+}$ HLA-DR^{+}	-0.03	0.81
**Lipid dysregulation**		
PE(36:3), pmol/mL	**-0.33**	**0.004 ***
PE(36:4), pmol/mL	**-0.37**	**0.001 ***
TG(16:1_18:1_18:1), pmol/mL	-0.20	0.08 **
GM3(d18:1/18:0), pmol/mL	**0.29**	**0.01 ***
HexCerd(d18:1/22:0), pmol/mL	0.20	0.08 **
HexCerd(d18:1/24:0), pmol/mL	0.18	0.12

ρ Spearman correlation coefficient. * denotes *p*-values <0.05. ** denotes *p*-values <0.10.

Supplementary Table 2a
Correlational analysis of immunometabolic markers

	%CD38/HLA-DR/CD4+		%CD38/HLA-DR/CD8+		sCD14 level, pg/mL		sCD163 level, ng/mL	
	ρ	*p*	ρ	*p*	ρ	*p*	ρ	*p*
**Metabolic dysregulation (Glut1 MFI)**								
Monocytes								
Total	-0.06	0.59	<0.001	1.00	0.13	0.26	**0.24**	**0.04** *
Non-classical	0.03	0.78	0.11	0.31	0.12	0.29	0.11	0.35
Intermediate	-0.08	0.47	-0.10	0.38	0.07	0.51	0.12	0.30
Classical	-0.04	0.74	0.02	0.85	0.08	0.46	0.16	0.15
**Mitochondrial Dysfunction ($DiOC_6(3)$ MFI)**								
Monocytes								
Total	-0.01	0.92	0.05	0.66	0.09	0.43	0.03	0.82
Non-classical	0.01	0.95	0.03	0.80	0.01	0.95	-0.01	0.94
Intermediate	0.01	0.94	0.06	0.57	0.08	0.46	0.02	0.87
Classical	0.04	0.71	0.01	0.93	0.05	0.64	-0.03	0.78

ρ Spearman correlation coefficient. * denotes *p*-values <0.05. ** denotes *p*-values <0.10.

Supplementary Table 2b
Correlational analysis of immunometabolic markers

**Metabolic dysregulation (Glut1 MFI)**	**Total**		**Non-classical**		**Intermediate**		**Classical**	
	ρ	*p*	ρ	*p*	ρ	*p*	ρ	*p*
**Mitochondrial Dysfunction ($DiOC_6(3)$ MFI)**								
Monocytes								
Total	-0.10	0.36	-0.06	0.61	-0.10	0.39	-0.05	0.66
Non-classical	-0.15	0.17	-0.10	0.36	-0.19	0.10	-0.05	0.66
Intermediate	-0.14	0.23	-0.06	0.57	-0.12	0.28	-0.10	0.39
Classical	**-0.27**	**0.01** *	-0.22	0.05 **	-0.19	0.09 **	**-0.24**	**0.03** *

ρ Spearman correlation coefficient. * denotes *p*-values <0.05. ** denotes *p*-values <0.10.

Supplementary Table 2c

Correlational analysis of immunometabolic markers and lipid subclasses

	PE(36:3)		PE(36:4)		TG (16:1_18:1_18:1)		GM3 (d18:1/18:0)		HexCerd (d18:1/22:0)		HexCerd (d18:1/24:0)	
	ρ	*p*	ρ	*p*	ρ	*p*	ρ	*p*	ρ	*p*	ρ	*p*
**Innate Immune Activation**												
sCD163 level, ng/mL	**0.30**	**0.01 ***	**0.25**	**0.03 ***	**0.31**	**0.01 ***	**-0.30**	**0.01 ***	-0.12	0.28	-0.12	0.30
sCD14 level, pg/mL	0.11	0.34	0.17	0.15	0.09	0.44	-0.03	0.77	0.002	0.99	0.018	0.88
**Metabolic Dysregulation (Glut1 MFI)**												
Monocytes												
Total	0.01	0.93	-0.01	0.90	0.01	0.90	-0.08	0.49	-0.08	0.47	-0.07	0.57
Non-classical	0.02	0.88	0.04	0.70	-0.03	0.82	-0.03	0.78	0.04	0.73	0.04	0.71
Intermediate	0.11	0.36	0.06	0.59	0.07	0.57	0.02	0.87	-0.07	0.55	-0.02	0.87
Classical	-0.01	0.91	-0.03	0.81	0.04	0.74	-0.11	0.36	-0.07	0.53	-0.07	0.57
**Mitochondrial Dysfunction ($DiOC_6$(3) MFI)**												
Monocytes												
Total	-0.02	0.85	-0.05	0.68	-0.14	0.22	0.16	0.18	0.07	0.56	0.06	0.60
Non-classical	-0.06	0.60	-0.08	0.50	-0.13	0.25	0.13	0.26	0.08	0.47	0.07	0.53
Intermediate	-0.01	0.92	-0.04	0.72	-0.12	0.30	0.16	0.16	0.07	0.57	0.06	0.63
Classical	0.03	0.77	-0.01	0.90	-0.13	0.27	0.17	0.14	-0.01	0.91	-0.04	0.74
**Adaptive Immune Activation**												
% $CD4^+$ $CD38^+$ $HLA\text{-}DR^+$	**0.24**	**0.04 ***	0.14	0.23	0.16	0.17	-0.11	0.34	-0.02	0.87	-0.05	0.66
% $CD8^+$ $CD38^+$ $HLA\text{-}DR^+$	**0.26**	**0.03 ***	0.19	0.10	0.21	0.07 **	-0.16	0.17	0.002	0.98	-0.04	0.72

ρ Spearman correlation coefficient. * denotes *p*-values <0.05. ** denotes *p*-values <0.10.
